# An Integrated NMR, LC-DAD-MS, LC-QTOF Metabolomic Characterization of *Sartoria hedysaroides*: Correlation of Antioxidant and Enzyme Inhibitory Activity with Chemical Composition by Multivariate Data Analysis

**DOI:** 10.3390/antiox11010110

**Published:** 2022-01-04

**Authors:** Stefano Dall’Acqua, Stefania Sut, Kouadio Ibrahime Sinan, Gokhan Zengin, Irene Ferrarese, Gregorio Peron, Evren Yildiztugay, Carene Picot-Allain, Mohamad Fawzi Mahomoodally

**Affiliations:** 1 Department of Pharmaceutical and Pharmacological Sciences, University of Padova, Via Marzolo 5, 35131 Padova, Italy; stefania.sut@unipd.it (S.S.); irene.ferrarese@unipd.it (I.F.); gregorio.peron@unive.it (G.P.); 2 Department of Biology, Science Faculty, Selcuk University, Konya 42130, Turkey; sinankouadio@gmail.com; 3 Department of Biotechnology, Science Faculty, Selcuk University, Konya 42130, Turkey; eytugay@gmail.com; 4 Department of Health Sciences, Faculty of Medicine and Health Sciences, University of Mauritius, Réduit 230, Mauritius; picotcarene@yahoo.com

**Keywords:** *Sartoria*, antioxidant, enzyme inhibition, coumarins, Turkey

## Abstract

*Sartoria hedysaroides* Boiss and Heldr. (Fabaceae) is an endemic plant of Turkey that has received little scientific consideration so far. In the present study, the chemical profiles of extracts from the aerial part and roots of *S. hedysaroides* obtained using solvents with different polarities were analyzed combining integrated NMR, LC-DAD-MS^n^, and LC-QTOF methods. In vitro antioxidant and enzyme inhibitory activities were evaluated, and the results were combined with chemical data using multivariate approaches. Phenolic acids, flavonoids, ellagitannins, and coumarins were identified and quantified in the extracts of aerial part and roots. Methanolic extract of *S. hedysaroides* aerial part showed the highest phenolic content and the highest antioxidant activity and cupric ion reducing antioxidant capacity. Dichloromethane extract of *S. hedysaroides* roots showed the highest inhibition of butyryl cholinesterase, while methanolic extract of *S. hedysaroides* aerial part was the most active tyrosinase inhibitor. Multivariate data analysis allowed us to observe a good correlation between phenolic compounds, especially caffeoylquinic derivatives and flavonoids and the antioxidant activity of extracts. Acetylcholinesterase inhibition was correlated with the presence of caffeoylquinic acids and coumarins. Overall, the present study appraised the biological potential of understudied *S. hedysaroides*, and provided a comprehensive approach combining metabolomic characterization of plant material and multivariate data analysis for the correlation of chemical data with results from multi-target biological assays.

## 1. Introduction

Nowadays, the use of medicinal plants is increasing not only among people having little access to modern healthcare, but also by urban citizens from developed countries [1]. This trend has been observed concurrently with the increase in the number of scientific articles reporting the benefits of herbal extracts. Several studies have attempted to appraise the benefits of endemic species in an endeavor to valorize the biological diversity of a specific Region. Turkey has a rich floral biodiversity of approximately 11,000 taxa, of which 1280 were reported to be traditionally used for medicinal purposes [2].

The Fabaceae family contains over 490 species, many of which have ethnopharmacological importance due to their peculiar chemical constituents, which can promote the healthy functioning of bodily systems [3,4]. Several species from the Fabaceae family have been documented in traditional medicine by local communities in Turkey for the management of various diseases [5,6]. *Sartoria hedysaroides* Boiss and Heldr. (Fabaceae), endemic to Turkey [7], has received little scientific consideration up to now. A previous study reported that acetone, ethanol, and methanol extracts of *S. hedysaroides* possess low antimicrobial activity [8].

The quest for novel therapeutical agents to treat ailments afflicting the world population is fueled by the need for more efficient and safe compounds. Plants have a wide range of chemical compounds possessing a multitude of biological properties. As such, studies have revealed the modulatory effect of phytochemicals on specific enzymes targeted on the treatment of Alzheimer’s disease, type 2 diabetes, and hyperpigmentation disorders. Furthermore, assessment of their antioxidant activity is essential owing to the key role of oxidative stress in the onset/progression of these conditions [9,10,11]. In this view, this study attempted to assess the multi-target biological activity of understudied *S. hedysaroides* as well as to furnish a comprehensive phytochemical characterization of extracts obtained by both the aerial part and roots by using solvents at different polarities.

## 2. Materials and Methods

### 2.1. Plant Material and Extraction

*S. hedysaroides* was collected in a field study on July 2019 (between Taskent and Alanya, Konya, 15 km, 1760 m). Plant materials were identified by Dr. Evren Yıldıztugay and a voucher specimen (EY-3031) was deposited in the Department of Biology, Faculty of Science, Selcuk University, Turkey. The aerial part and roots were carefully separated and then the samples were dried in shade condition for one week. Dried plant materials were grounded by using a laboratory mill and they were stored in dark conditions.

Different solvents were used to obtain extracts. These were dichloromethane (DCM), ethyl acetate (EA), methanol (MeOH) and water. The extracts obtained with organic solvent were prepared by maceration. For this purpose, plant materials (10 g) were macerated with 200 mL of solvents in a magnetic stirrer for 24 h at room temperature. After that, the mixture was filtered and then solvents were removed by using a rotary evaporator. With regard to water extract, the traditional infusion technique was used, namely, plant materials (10 g) were kept in 200 mL of boiled water for 15 min. Then, the mixture was filtered and lyophilized. All extracts were stored at 4 °C until analysis.

### 2.2. NMR Analysis

The NMR analysis was performed on a Bruker Avance 3 spectrometer operating at 400 MHz for ^1^H. Extracts (20 mg) of aerial or root extract were dissolved in deuterated methanol and filtered; then, the liquid was used for the acquisition of spectra. Standard Bruker pulse sequences were used measuring ^1^H, HSQC-DEPT, HMBC, and COSY, spectra.

### 2.3. LC-DAD-ESI-MS^n^

The analysis was performed on an Agilent 1260 chromatograph equipped with a 1260 series autosampler and a 1260 diode array detector (DAD). The chromatograph was also linked to a Varian MS500 mass spectrometer equipped with Electrospray ion source (ESI). Gradient elution was used with the following eluents: 0.1% formic acid in water (A), acetonitrile (B), and methanol (C). Gradient started with 98% A and 2% B and was kept for 1 min in isocratic condition. Then, in 10 min, it reached 90% A, 8% B, and 2% C. In 20 min, it reached 70% A, 28% B, and 2% C, and it was maintained in isocratic conditions for 5 min. At 30 min, the composition was 0% A, 80% B, and 20% C, and it was maintained for 9 min. Finally, solvent composition was restored to the initial condition, and equilibration time was 4 min. The column used was an Agilent Eclipse C-18 (3.5 µm, 3.0 × 150 mm). Flow rate was 0.4 mL/min. The DAD was set to acquire spectra from 200–400 nm. MS data were acquired in both positive (ESI(+)) and negative (ESI(−)) ion modes. Nebulizer gas pressure was 35 psi, drying gas pressure was 15 psi, and the temperature of the drying gas was 300 °C. Capillary voltage was set to 80 V, and RF was 85%. Fragmentation of the main ionic species was obtained using the tdds^®^ instrument utility.

Dried extracts were weighted (50.1 mg ± 0.1) and dissolved in 10 mL of methanol by using an ultrasound bath for 5 min. Liquids were centrifuged at 13,000 rpm for 15 min and then used for analysis.

Quantification of extracted phytoconstituents was achieved by using DAD, and solutions of different reference compounds were used to create appropriate calibration curves. For the quantification of hydroxycinnamic acid derivatives, chlorogenic acid was used as the reference compound, and the calibration curve built at 330 nm in the concentration range 100–1 µg/mL was *y* = 45.125*x* + 0.116 (R^2^ = 0.9989). For the quantification of flavonols, rutin solutions in the concentration range 100–1 µg/mL were used, and the calibration curve at 350 nm was *y* = 25.3*x* + 0.55 (R^2^ = 0.9991). Taxifolin was used for the quantification of taxifolin derivatives, and the calibration curve was built using solutions in the concentration range of 120–0.61 µg/mL. The calibration curve at 280 nm was *y* = 9.62*x* + 0.0321 (R^2^ = 0.9989). Coumarin was used for the quantification of coumarin and related derivatives, using solutions in the concentration range 120–1.20 µg/mL. The calibration curve at 254 nm was *y* = 3.18*x* + 0.033 (R^2^ = 0.9989). Finally, ellagic acid was used for the quantification of ellagic acid derivatives, and solutions were prepared in the concentration range 150–0.3 µg/mL. The calibration curve at 280 nm was *y* = 75.12*x* + 1.35 (R^2^ = 0.9989).

### 2.4. UPLC-QTOF Analysis

UPLC-QTOF analysis of *Sartoria* extracts was performed by using a Waters Acquity UPLC system coupled to a Waters Xevo G2 QTOF MS detector. As stationary phase, an Agilent Zorbax Eclipse Plus C18 (2.1 × 50 mm, 1.8 µm) column was used, which was maintained at 40 °C. A mixture of water with 1% formic acid (A) and methanol with 1% formic acid (B) was used as mobile phase. The elution gradient was as follows: 0–1 min, 98% A; 11 min, 15% A; 16 min, 0% A; 20 min, 0% A; 21 min, 98% A; 24 min, 98% A. The flow rate was 0.3 mL/min, and the injection volume was 2 μL. MS data were acquired in both ESI(+) and ESI(−) in the mass range 50–2000 Da. The sampling cone voltage was adjusted at 40 V, the source offset at 80 V. The capillary voltage was adjusted to 3.5 KV. The nebulizer gas used was N_2_ at a flow rate of 800 L/h. The desolvation temperature was 450 °C. The mass accuracy and reproducibility were maintained by infusing lock mass (leucine–enkephalin, [M + H]^+^ = 556.2771 *m*/*z*, and [M − H]^−^ = 554.2620 *m*/*z*) through Lockspray at a flow rate of 20 μL/min. The *m*/*z* value of all acquired spectra was automatically corrected during acquisition based on lock mass. MS^e^ experiment was simultaneously performed to collect structural information, setting the collision energy at 30 V.

### 2.5. Antioxidant and Enzyme Inhibitory Assays

In the current work, the antioxidant effects of the tested extracts were detected by different assays [12]. The assays were 1,1-diphenyl-2-picrylhydrazyl (DPPH) and 2,2′-azino-bis(3-ethylbenzothiazoline) 6-sulfonic acid (ABTS) radical scavenging, cupric ion reducing antioxidant capacity (CUPRAC), ferric ion reducing antioxidant power (FRAP), metal chelating ability (MCA), and phosphomolybdenum assay (PDA). For DPPH, ABTS, CUPRAC, and FRAP, assays data were expressed as mg Trolox equivalents (TE)/g extract, whereas in MCA and PDA, mg EDTA equivalents (EDTAE)/g extract and mmol TE/g extract were used, respectively. Total phenolic content (TPC) and total flavonoid content (TFC) were determined as previously described [12,13], and expressed as mg gallic acid equivalents (GAE)/g extract (TPC) and mg rutin equivalents (RE)/g extract (TFC). The experimental parts for acetylcholinesterase, butyrylcholinesterase, tyrosinase, amylase, and glucosidase assays were previously provided. Galathamine was used as a positive control in cholinesterase assays and data were evaluated as mg galanthamine equivalents (GALAE)/g extract. Kojic acid was used as a standard inhibitor in tyrosinase inhibitory assay and the results were expressed as mg kojic acid equivalents (KAE)/g extract [12,13]. Acarbose was selected as inhibitors for amylase and glucosidase inhibtory assays and the results are given as mmol acarbose equivalents (ACAE)/g extract. All experimental details of the assays are reported in the supplementary material.

### 2.6. Data Analysis

The parametric One-way ANOVA was carried out to evaluate the significance of differences between the extracts. Principal component analysis (PCA) and Clustered Image Map were performed to explore the similarity between the extracts. Data was standardized before achieving the PCA, and Clustered Image Map was performed using “Euclidean norm” and “Ward linkage”. Then, based on Pearson’s correlation coefficients, the relationship between the studied biological activities and the quantified phytochemical content was determined. The R packages FactoMineR was used for all calculations.

Correlation analysis among phytoconstituents of *S. hedysaroides* extracts and biological activities was performed using the Spearman rank correlation test. Data pre-processing included the removal of variables with more than 80% missing values, the imputation of the remaining missing values using the KNN algorithm, and finally data normalization by means of *log* transformation and Pareto scaling. Analysis was performed using the Metaboanalyst v. 4.0 platform [14].

## 3. Results and Discussion

### 3.1. Phytochemical Characterization of S. hedysaroides Extracts

Table 1 summarizes the TPC and TFC of the different extracts from the aerial part and roots of *S. hedysaroides*. In general, the extracts prepared from the aerial part possessed higher TPC and TFC compared with those obtained from the roots. Among the aerial part extracts, the methanolic one showed highest TPC (67.64 mg GAE/g) and TFC (92.03 mg RE/g). Assessing the TPC and TFC provides an indication of the antioxidant potential of herbal extracts and is thus routinely performed for the determination of their bioactivity.

**Figure 1:**
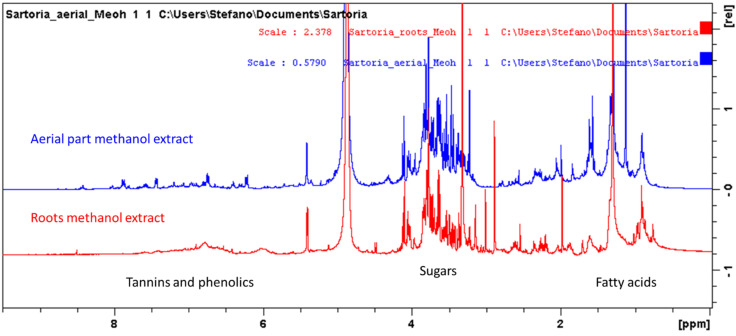
^1^H-NMR of methanol extracts from *S. hedysaroides* aerial part and roots. Identified classes of compounds are indicated.

As a screening technique, ^1^H-NMR was used to assess the presence of main plant compounds in the extracts obtained from roots and the aerial part. A fingerprint was obtained selecting methanol as the solvent. This because it can dissolve both hydrophilic and lipophilic compounds, thus providing the opportunity to observe a large number of different metabolites. Comparing the ^1^H-NMR of methanol extracts of leaves and roots (Figure 1), it is clear that both extracts presented signals ascribable to fatty acids as well as sugar derivatives. Considering the aromatic compounds, more evident signals were visible in the leaves extract, while root sample presented broad signals in the aromatic part that may suggest the presence of tannins or polymeric phenolics (Figure 1). Thus, for the further LC-DAD-MS analysis, phenolics and tannins were selected as the target compounds.

**Figure 2:**
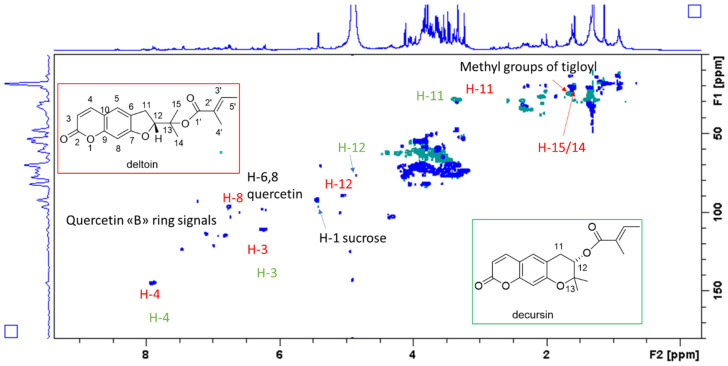
HSQC-DEPT spectrum of methanol extract from *Sartoria* aerial part. Structures of the two main constituents, namely deltoin and decursin, are highlighted. Main assignments are also indicated with red and green colors, respectively.

Further investigations were obtained acquiring 2D measurements on the aerial part extract, in particular combining data from HSQC-DEPT, HMBC, COSY, and TOCSY spectra. A series of diagnostic signals can support the presence of a notable amount of linear dihydrofuranocoumarin derivatives (Figure 2) [15,16]. The protons of the double bond of the lactone ring were observed as doublets at δ 7.85 (H-4) and 6.23 (H-3). These signals showed a correlation in the COSY spectrum (isolate spin system), and diagnostic HMBC were observed from H-4 with C-2 (δ 163.0), C-9 (δ 154.0), and C-5 (δ 123.0), supporting the presence of a coumarin with non-oxygenated C-8 position. In fact, if an oxygenated substituent would be present in C-8, then a chemical shift of C-9 would be shifted at δ 140. This observation was confirmed by the assignment of the H-8 to a signal at δ 6.55 that directly correlated in HSQC with a carbon at δ 96.0, and showed diagnostic long-range HMBC correlations with carbon resonances C-6 (δ 127.0), C-7 (163.0), C-10 (112.6), and C-9. The value of the chemical shift of position 7 supports a single oxygenation of the ring, while the HMBC observed from H-5 with carbon resonance at δ 28.7 (C-11) supports the presence of an aliphatic substituent. COSY spectrum from H-11 (δ 3.36-3.30) showed a correlation only with H-12 (δ 4.90 overlapped to methanol OH signal), and with this latter the spin system looked to stop, thus suggesting the presence of a dihydrofuran ring linked to positions 6,7 of the coumarin. Further signals ascribable to this compound were the quaternary methyl groups at δ 1.64 and 1.59 directly connected with carbon resonances at δ 20.4 and 20.9. Long-range HMBC correlations were observed from these two methyl groups with C-13 (δ 82.0) and C-12 (δ 89.0), suggesting the presence of a hydroxy-isopropyl moiety. Esterification could be deduced from the chemical shift of the position C-13. Signals supporting the presence of tiglic acid residue were observed, namely the proton at δ 6.80, directly linked to a carbon resonance at δ 138.1, and two methyl groups at δ 1.77 and 1.65, directly linked to carbon resonances at δ 16.5 and 14.0. These latter C-4′ and C-5′ showed HMBC correlations with carbons at δ 129.7 and 138.1, respectively. The proton signal of C-4′ at δ 1.65 showed diagnostic HMBC with the carbonyl function at δ 167.8 (C-1′). NOESY correlations observed from the signal at δ 6.40 (H-3′) and the methyl group at δ 4.61 confirmed the presence of tigloyl moiety. The evaluation of all these data indicated that some coumarin derivatives were present in the extract and that one of the main compounds can be ascribed to the structure of deltoin. Signals supporting the structure of the isomer decursin were also observed; in fact, the two isomers presented very similar ^1^H-NMR spectra with different chemical shift of positions 11 and 12 due to the presence of a pyran ring in decursin (see Table 2).

Derivatives corresponding to the angular dihydrofurocoumarins were not detectable with NMR. In fact, in the case of columbianidin or derivatives, we should observe *ortho* coupling proton signals of positions 5 and 6 that were not prevalent or observable in the spectrum of the crude extract. Minor signals can be ascribed to linear furocoumarins, namely the H-11 at δ 6.70 and the H-12 at δ 7.60 directly linked to carbon resonances at δ 104 and 145 respectively. However, these compounds were present in lower abundance compared to deltoin.

Signals supporting the presence of flavonoids could be observed and are reported in Table 1. These signals are less intense than coumarin ones and their correlations in HMBC and COSY were in part superimposed to other signals. Hence, they were not sufficiently clear to assess a single compound identification.

**Figure 3:**
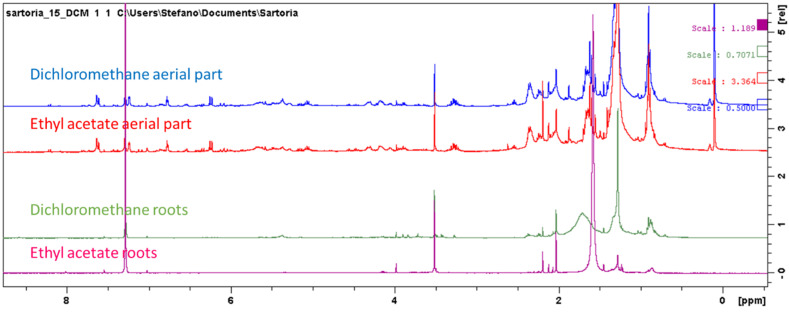
Superimposition of the ^1^H-NMR spectra of lipophilic extracts of the aerial part and roots.

Further compounds that were identified are sucrose (Table 1) and fatty acids. Coumarin derivatives were present only in the aerial part. Figure 1 and Figure 3 show superimposition of the spectra of the different extracts, and it can be observed that signals ascribable to the aromatic and olefinic portions of coumarin derivatives were not detected in root samples. In lipophilic extracts, as expected, signals supporting the presence of fatty acids were observed.

Due to the results obtained by NMR fingerprinting, LC-DAD-ESI-MS^n^ and LC-QTOF analyses were performed on *Sartoria* extracts by operating both in positive and negative ion modes. Ion trap analysis allowed us to observe the fragmentation pathways of compounds, and hence to collect qualitative data about their structures. On the other hand, high resolution mass spectra obtained with Q-TOF allowed us to calculate the molecular formula of each constituent. Overall, the combination of data obtained from the two platforms allowed us to perform the putative identification of the chemical constituents in the extracts (level 2 identification, as proposed by the metabolomics standards initiative [17,18]) (Table 3, Table 4 and Table 5).

Three main classes of compounds were detected. Identified phytocompounds could be grouped in phenolic and organic acids (comprising flavonoids and hydroxycinnamic and hydroxybenzoic acids derivatives), ellagic acid derivatives, and coumarins.

**Figure 4:**
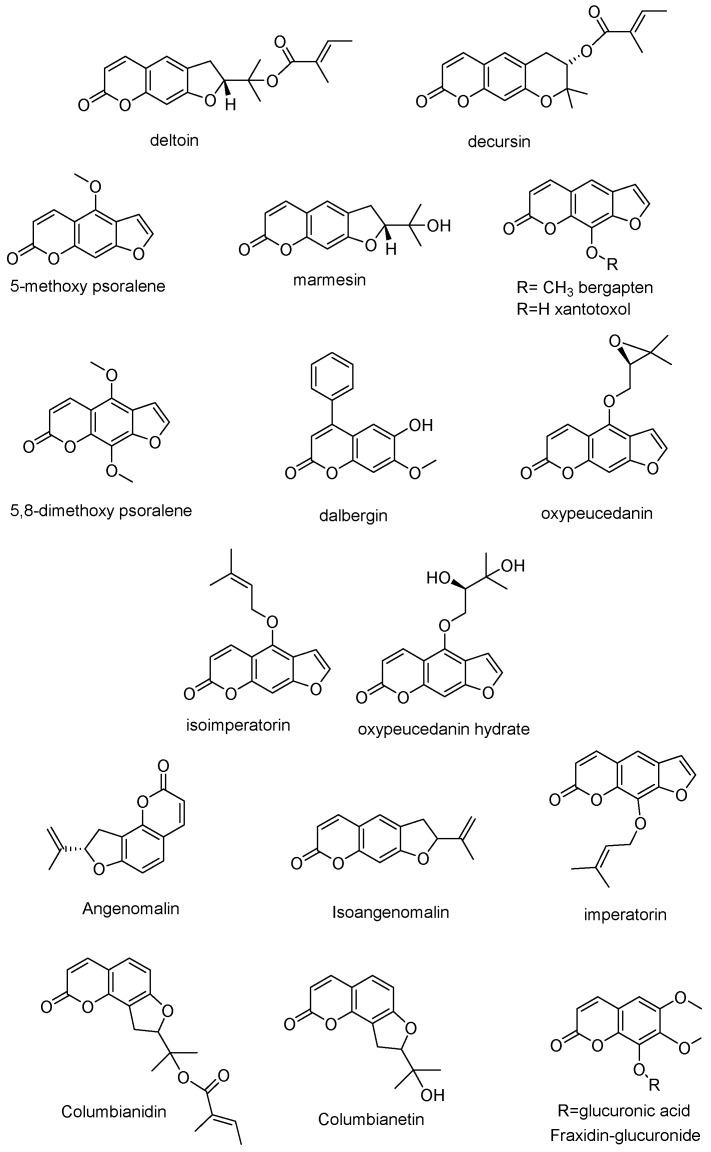
Chemical structures of coumarins identified in the extracts.

Identification of compounds was achieved by comparison of accurate MS values as well as by evaluating MS^n^ fragmentation patterns obtained from ion trap analysis. Small organic acids were identified on the basis of their UV spectra, accurate MS and mass fragmentation, and were mainly quinic acid, protocatecuic acid hexoside, and vanillic acid hexoside. Quinic acid esters with hydroxycinnamic acid were identified based on their characteristic UV spectra obtained by DAD, showing a maximum of absorption at 315–330 nm. Furthermore, MS fragmentation in ion trap allowed us to observe losses of caffeic acid (−162 a.m.u.), p-coumaroyl (−146 a.m.u.), or feruloyl (−176 a.m.u.) moieties, and an ion corresponding to quinic acid (*m*/*z* 191). As reported in the literature [19,20], intensity of the ion species observed in MS^2^ and MS^3^ spectra allow the identification of the main isomers as exemplified for 3,5-dicaffeoylquinic and 4,5-dicaffeoylquinic acids (Table 3). Flavonoids were identified on the basis of their characteristic UV spectra and typical MS fragmentation [21,22]. Main derivatives were quercetin glycosides and taxifolin derivatives. Information on the glycosidation site of flavonoids could be obtained by the MS^n^ measurements [23]. Tannins formed by derivatives of ellagic acid were observed, and all presented a fragment ion at *m*/*z* 303 in ESI(+) MS/MS, corresponding to ellagic acid. Coumarin derivatives could be observed in positive ion mode [24,25], and characteristic fragmentation pathways supporting the loss of tygloyl moiety (-100 a.m.u.), CO (-28 a.m.u.), and CO_2_ (−44 a.m.u.) were detected. Furthermore, other losses (−56 a.m.u. and −42 a.m.u.) were also observed. Such fragmentations are compatible with structures of deltoin and columbianadin (with molecular ion [M + H]^+^ at *m*/*z* 329) [25]. The structure of deltoin was also confirmed by comparison of NMR data and by analysis of an authentic sample. Other classes of coumarins were also detected although in lower amounts, namely, linear furocoumarins as xantotoxin (*m*/*z* 217) and bergapten (*m*/*z* 217). All the identified coumarin derivatives are reported in Figure 4 and information about all the putatively identified constituents is reported in Table 5.

Quantification of plant secondary metabolites was performed with the use of appropriate reference compounds, namely, chlorogenic acid for phenolic and organic acids, rutin for flavonoids, ellagic acid for tannins, and coumarin for coumarin derivatives. Organic acids and phenolics were present in higher amounts in methanol and water extracts of aerial parts, and the most abundant compounds were rutin and 4-feruloylquinic acid. Obtained data are in agreement with TPC and TFC results (Table 1). Tannin derivatives were mostly detected in roots, and were mostly extracted by methanol (94.6 mg/g), followed by water (65.5 mg/g), dichloromethane (62.5 mg/g), and ethyl acetate (36.49 mg/g). Coumarin derivatives were mainly present in the aerial part, especially in the dichloromethane (132.8 mg/g) and methanol (105.0 mg/g) extracts.

### 3.2. Antioxidant Effects

Determination of the antioxidant potential of *S. hedysaroides* extracts was carried out using free radical scavenging assays, metal chelating assay, as well as reducing potential. From Table 6, it can be noted that the methanolic extracts of *S. hedysaroides* aerial part showed the highest radical scavenging potential (169.97 and 285.33 mg TE/g, for DPPH and ABTS assays, respectively). In fact, the role of free radicals in oxidative stress and consequent pathological conditions has been extensively reported in literature. In the quest of natural, safe radical scavengers, the use of rapid, simple, and cheap radical scavenging assays, such as DPPH and ABTS, is beneficial. Exploring different mechanisms by which natural compounds might exhibit antioxidant potential is imperative to understand their mechanism(s) of action. Results showing the ability of *S. hedysaroides* extracts to reduce colorless Fe^3+^-TPTZ to blue Fe^2+^-TPTZ complex in acidic condition, blue-green Cu^2+^-neocuproine to yellow Cu^+^-neocuproine complex, and Mo (VI) to Mo (V) are reported in Table 6. In both CUPRAC and FRAP assays, the methanolic extract of *S. hedysaroides* aerial part showed the highest reduction potential (CUPRAC: 279.23 mg TE/g; FRAP: 162.48 mg TE/g). However, the best reduction ability was observed for the ethyl acetate extract of aerial part (2.08 mmol TE/g) in the phosphomolybdenum assay. The metal chelating property of *S. hedysaroides* extracts was also assessed as a method to assess their antioxidant potential. The ability of natural compounds to chelate metal ions has been advocated as a method to suppress Fenton reaction and the consequent formation of free radicals. From Table 6, it can be noted that water extracts, which possessed the lowest TPC/TFC, showed the highest metal chelating potential. Moreover, the water extract of *S. hedysaroides* roots showed higher activity as compared with the aerial part. To the best of our knowledge, there are no data on antioxidant properties of *S. hedysaroides*, and therefore our results provide a valuable contribution to the scientific literature regarding this plant species.

**Figure 5:**
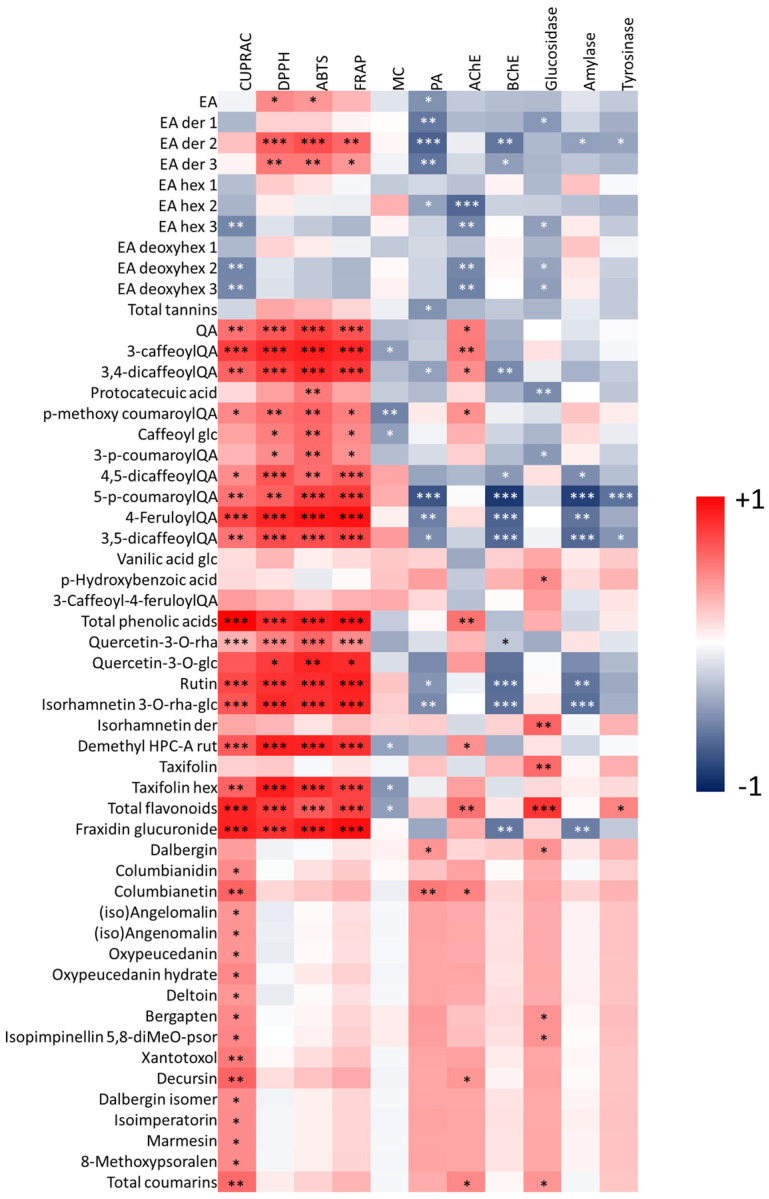
Correlation heatmap showing positive (red color) and negative (blue color) correlations (−1 < Pearson’s r < 1) between *Sartoria* phytoconstituents and biological activities. Der: derivative; EA, ellagic acid; diMeO: di methoxy-; hex, hexoside; HPC-A, hedysarimpterocarpene-A; MC, metal chelating activity; PA: phosphomolybdenum; psor: psoralene; QA, quinic; rha, rhamnoside; rut, rutinoside. *: *p*-value < 0.05; **: *p*-value < 0.01; ***: *p*-value < 0.001.

In a structure-ability relationship framework, the heatmap correlation analysis showed that some chemical components mainly contribute to the observed antioxidant abilities of the tested extracts (Figure 5). For example, quinic acid, caffeoylquinic acid, and quercetin derivatives exhibited a strong correlation with the antioxidant properties. In accordance with our approach, these compounds were reported by several researchers as strong antioxidants. For example, Hung et al. [26] and Cao et al. [27] reported the remarkable antioxidant properties of six caffeoylquinic acid derivatives. Especially, the attachment of caffeoyl groups into quinic acid and the presence of hydroxyl groups could improve the antioxidant properties [27]. Similar to phenolic acids, the number of hydroxyl groups in quercetin moiety greatly enhance its antioxidant properties [28].

### 3.3. Inhibitory Activity of S. hedysaroides Extracts against Selected Enzymes

The inhibition of specific enzymes has been claimed to be a successful therapeutic strategy for the management of several ailments. In this sense, the search for novel, effective and safe enzyme inhibitors is one of the most attractive topics in pharmaceutical research. In the present study, we examined the inhibitory effects of *S. hedysaroides* aerial and root extracts against acetyl- and butyryl-cholinesterase, tyrosinase, α-amylase, and α-glucosidase (Table 7). The water extracts of *S. hedysaroides* aerial part and roots showed no activity against both cholinesterase enzymes. Low concentration of the neurotransmitter acetylcholine in the brain due to hydrolysis by cholinesterases has been associated with Alzheimer’s disease [29]. Therefore, administration of cholinesterase inhibitors is regarded as a therapy for Alzheimer’s disease. Multi-target molecules showing activity against both acetyl- and butyryl- cholinesterase have been suggested as an effective pharmacotherapy strategy to manage Alzheimer’s disease [30]. The galantamine equivalent values for acetylcholinesterase were lower as compared with butyrylcholinesterase, implying that the extracts exhibited higher inhibition of butyrylcholinesterase. The DCM extract of *S. hedysaroides* roots showed the highest activity against butyrylcholinesterase (6.03 mg GALAE/g), followed by DCM extract of the aerial part (5.96 mg GALAE/g). Regarding tyrosinase inhibition, both methanolic extracts showed pronounced activity, but methanolic extract of aerial part showed higher activity. Tyrosinase is responsible for melanogenesis and abnormal accumulation of melanin, which causes various dermatological disorders [31]. As such, tyrosinase inhibitors find application in the management of hyperpigmentation conditions, such as melasma, freckles, and acne scars. In general, a low inhibitory activity of *Sartoria* extracts was observed on α-amylase (0.10–1.08 mmol ACAE/g) and α-glucosidase (0.02–1.02 mmol ACAE/g). A literature search did not reveal any data on the enzyme inhibitory effects of *S. hedysaroides*. As a chemical insight, the heatmap reported in Figure 5 shows the relationship between chemical structures and bioactivity. We observed a moderate correlation between coumarins and AchE inhibition. This was expected due to the literature data, which show the significant but moderate anti-AchE activity of different coumarin derivatives [32,33]. Additionally, the presence of 3-caffeoylquinic acid was correlated with the inhibition of AchE. A previously published paper reports that a potential efficient binding between 3-caffeoylquinic acid and AchE can be established in silico [34]. Furthermore, other works revealed that 3-caffeoylquinic acid is able to inhibit both AchE and BchE [35,36]. A previous investigation on the ability of 3-caffeoylquinic acid to inhibit these enzymes led to the measurement of IC_50_ values of 8.0 µg/mL and 6.3 µg/mL for AchE and BchE, respectively. The authors concluded that the inhibitory properties of caffeic acid are higher than 3-caffeoylquinic acid, suggesting that the esterification with quinic acid reduces the ability of the compound to interact with enzymes. In fact, for caffeic acid the IC_50_ values against AchE and BchE were respectively 4.2 µg/mL and 5.6 µg/mL [37]. To comment on these literature data, we must consider that the molecular weight of 3-caffeoylquinic acid is 354 Da and that of caffeic acid is 180 Da, while IC_50_ values for 3-caffeoylquinic acid on AchE and BchE are 22.6 µM and 17.7 µM, respectively, and 23.3 µM and 70 µM for caffeic acid. These numbers suggest that, at molar equivalence, the bioactivity is higher for the quinic ester than for the free derivative [37]. Besides cholinesterases, taxifolin and isorhamnetin showed a good correlation with the glucosidase inhibitory ability. Our findings are also supported by other studies [38,39] that have previously reported that the flavonoids show promising glucosidase inhibitory effects. In addition to the structure-activity relationship, studying the effect of the extract may also result in potential interactions (antagonistic or synergetic) of the different phytochemicals leading to different activity on the enzymes. Thus, further studies should be performed using isolated components of *S. hedysaroides* on the different considered enzymes.

### 3.4. Multivariate Analysis of Biological Data

To assess possible (dis)similarity trends among the different extracts, an exploratory multivariate analysis was performed, considering the data obtained from bioassays. The use of univariate approaches was potentially insufficient to emphasize all relevant information present in the data set.

**Figure 6:**
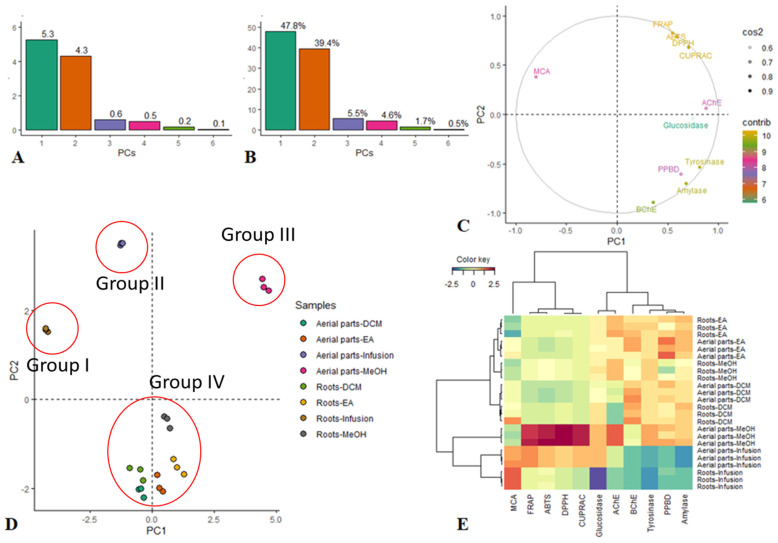
Exploratory multivariate analysis of the biological activities of *Sartoria hedysaroides*. (**A**,**B**): Eigenvalue and percentage of explained variances of principal components. (**C**): Circle of correlation showing the contribution of biological activities to the first two principal components. (**D**): Projection of the samples on the factorial plane PC1 vs PC2. Groups, as reported in the text, are indicated in the Figure with red circles. (**E**): Clustered Image Map (Ward linkage, Euclidean distance). Red color: high activity. Blue color: weak activity.

Firstly, principal component analysis was performed to reduce the dimensionality of the data, and results are reported in Figure 6. Both the eigenvalue and the percentage of variances were determined for the number of principal components [40]. According to the Kaiser rule, only the components with eigenvalues above one must be retained. In addition, given that the total amount of variance that should be considered is often subjective, the components accounting for at least 80% of variabilities were accepted. Based on both the above-mentioned criteria, the first two principal components accounting for 87.2% of variability were accepted (Figure 6A,B). The combination of the remaining components contributed 12.8%. In Figure 6, the contribution of each biological activity on the PCs can be observed. The PC1 was highly loaded by activities on AChE, MCA, tyrosinase, and glucosidase, suggesting that PC1 discriminated the samples predominantly according to them. The PC2 was strongly loaded for antioxidant capacities (ABTS, DPPH, FRAP, CUPRAC) and activity on BChE and amylase; indeed, PC2 may be appointed as an antioxidant, as anti-BChE and anti-amylase capacities disparity among the extracts. By reference to the PCA score plot (Figure 6D), it can be observed that the extracts were separated into four groups. Groups I and II, composed respectively by water extracts of the aerial part and roots, were away from each other and ranged in the negative and positive sides of PC1 and PC2, respectively. Group III containing the methanol extract of aerial part was separated from the first two groups along PC1. Roots-EA, aerial part-EA, roots-DCM, aerial part-DCM, and roots-MeOH extracts, close together on the negative side of PC2, formed Group IV. Considering the chemical data obtained by NMR and LC-MS approaches (summarized in Table 3), we can observe that water extract of aerial part (Group I) mostly contained phenolics (hydroxycinnamic acids and flavonoids) in a total amount of 58 mg/g of dry extract. The water extract of roots (Group II) was instead richer in tannins (66 mg/g). On the other hand, the methanol extract of the aerial part presented a higher content of coumarins and phenolics (hydroxicinnamic acids and flavonoids) with a content of 105 mg/g and 42 mg/g of dried extract, respectively. The extracts belonging to the Group IV presented an intermediate content of all the identified compounds, with an abundance of different classes of compounds: as an example, the DCM roots extract presented high content in tannins (63 mg/g) and coumarins (133 mg/g).

The cluster image map provided an indication that Group III (methanol extract of the aerial part) and to a lesser extent Group I (water extract of aerial part) exhibited a relatively higher antioxidant capacity than other extracts (Figure 6E). ABTS, DPPH, FRAP, and CUPRAC were found to be positively correlated to total phenols and total flavonoids content (Table 8). This highly positive correlation supports the hypothesis that flavonoids and phenolic compounds are significant contributors to the antioxidant properties of *S. hedysaroides*. For the sake of argument, the largest concentration of phenolic and flavonoid compounds was found in methanol and water extracts of aerial part. In addition, chromatographic analysis demonstrated that *S. hedysaroides* extracts, in particular methanol and water extracts of the aerial part, were rich in well-known phenolic antioxidants, including distinctive compounds such as hydroxycinnamic derivatives and flavonoids. The antioxidant capacities of phenolic compounds arise from their redox properties, which allow them to act as metal chelators, reducing agents, single oxygen scavengers, and hydrogen donators [41]. Furthermore, the heatmap reported in Figure 6 clearly shows a strong positive correlation between hydroxycinnamic acids and flavonoids and the results from antioxidant assays. A strong correlation was observed with rutin and 4-feruloylquinic acid, which are the most abundant compounds in methanol and water extracts of the aerial part. In the coumarin group, only fraxidin-glucuronide presented a strong positive correlation with those bioassays, being this compound that is most abundant in the water extract of the aerial part. Group II (water extract of roots) showed the lowest anti-enzymatic capacities, despite being found to possess excellent chelating ability against ferrous ion. Group IV, comprising all the other extracts (DCM and ethyl acetate extracts of the aerial part; DCM, ethyl acetate, and methanol extracts of roots), had very similar responses in the biological assays. Considering the chemical constituents of these extracts (as reported in Table 3), roots extracts mostly contained phenolics (hydroxycinnamic acids and flavonoids and) and tannins, with limited the amount of coumarins.

## 4. Conclusions

A comprehensive chemical analysis of the aerial part and roots extracts from *S. hedysaroides* endemic to Turkey was reported here for the first time. Aerial part extracts obtained with different solvents were all rich in coumarins, while only water and methanol were able to extract significant amounts of hydroxycinnamic derivatives, flavonoids, and tannins. The root extracts presented high tannin contents. Multivariate data analyses were performed to correlate chemical information with biological results obtained from antioxidant and enzyme inhibitory assays. Methanol and water extracts of the aerial part presented the most promising results in bioassays, mainly related to their antioxidant activities. Multivariate data analysis allowed us to highlight a positive correlation between the antioxidant activity of extracts and the presence of caffeoyl esters and flavonoids. The less polar extracts of *S. hedysaroides* aerial part and roots showed higher inhibition of acetyl- and butyryl- cholinesterases, and the correlation heatmap indicated a positive correlation of this effect with the content of coumarins (present only in the aerial part), caffeoyl esters, and quinic acid derivatives, which were present in both aerial part and roots. Overall, the obtained data revealed the potential usefulness of *S. hedysaroides* as source of antioxidants and bioactive compounds. This plant species can be considered as a potential raw material for the development of new applications in the pharmaceutical industry. However, more research is needed to explore the toxic properties of *S. hedysaroides* through cell or animal studies.

## Figures and Tables

**Table 1:** Total phenolic and flavonoid contents of *S. hedysaroides* extracts.

	Solvents	TPC (mg GAE/g) *	TFC (mg RE/g) *
Aerial parts	DCM	24.70 ± 0.59 ^e^	8.30 ± 0.55 ^e^
	EA	29.17 ± 0.10 ^c^	8.33 ± 0.40 ^e^
	MeOH	67.64 ± 0.63 ^a^	92.03 ± 0.43 ^a^
	Infusion	45.33 ± 0.21 ^b^	59.54 ± 0.80 ^b^
Roots	DCM	22.17 ± 0.06 ^f^	15.06 ± 0.54 ^d^
	EA	29.82 ± 0.36 ^c^	16.91 ± 0.24 ^c^
	MeOH	27.62 ± 0.17 ^d^	16.23 ± 0.09 ^cd^
	Infusion	24.56 ± 0.14 ^e^	1.27 ± 0.22 ^f^

* Values are reported as means ± SD. DCM: Dicholoromethane; EA: Ethyl acetate; MeOH: Methanol; TPC: Total phenolic content; TFC: Total flavonoid content; GAE: Gallic acid equivalent; RE: Rutin equivalent. Different superscripts indicate significant differences in the extracts (*p* < 0.05).

**Table 2:** NMR assignments of the main constituents detected in the methanol extract of *S. hedysaroides* aerial part.

Class of Compounds	δH	δC	HMBC	COSY/NOESY
Deltoin and decursin				
2	-	162	-	-
3	6.23	111.0	163 112.6	7.85 H-4 COSY
4	7.85	144.0	163 154 123	6.20 H-3 COSY
5	7.44	123.0	144 163 154	-
6	-	127	-	-
7	-	164	-	-
8	6.75	96.0	163 154 127 113	-
9	-	154	-	-
10	-	113	-	-
11 Deltoin	3.36–3.30	28.7	127 89 82	4.90 COSY
12 Deltoin	4.90 (under methanol)	89		3.30–3.36 COSY
11 Decursin	3.40	28.0	127 89 82	4.87 COSY
12 Decursin	4.86 (under methanol)	77		3.30–3.36 COSY
14	1.59	20.9	82 89 20	
15	1.63	20.5	82 89 20	
Tigloyl moiety of deltoin and decursin				
1′	-	167	-	-
2′	-	130	-	-
3′	6.40	137	167 137 130	
4′	1.60	20 superimposed	167 137 130	
5′	1.64	20 superimposed	137 130	
Furocumarins				
11	6.68–6.60	106–104	-	-
12	7.60–7.65	145	158 116	-
Flavonols				
6, 8	6.40 6.30 6.19	99.1 96.5 92.5		
2′	7.68	126.5		
5′	6.73	115		
6′	7.25	114.5		
Sucrose				
1	5.30	92.8	-	-
2	3.55	76.4		
6	3.82	62.6	-	-
1′	3.67	76.4	-	-
3′	4.21 d	77.4	-	-

**Table 3:** Content of phenolic and organic acids identified in the extracts, expressed as mg/g of dry extract. MS data were acquired in negative ion mode (ESI(−)).

					Aerial				Roots			
Name	Formula (Neutral Compound)	Theorical *m*/*z* *	Exp *m*/*z* *	ppm	DCM	EA	MeOH	W	DCM	EA	MeOH	W
**Phenolics and Organic acid**												
Quinic acid	C7H12O6	191.0555	191.0565	−5.55	-	0.10	2.16	0.25	-	0.06	5.09	0.19
Caffeoyl glucose	C15H18O9	341.0872	341.0887	−4.66	-	-	0.09	-	-	0.03	0.01	0.03
3-p-Coumaroyl quinic acid	C13H16O9	337.0923	337.0937	−4.40	-	-	0.12	-	-	0.03	0.02	0.08
Protocatecuic acid hexoside	C16H18O9	315.0716	315.0731	−5.05	-	-	0.20	-	-	0.04	0.17	0.30
p-Methoxy coumaroyl quinic acid	C17H20O8	351.1079	351.1095	−4.83	-	-	0.46	-	-	0.14	0.02	0.04
Quercetin-3-O-rhamnoside	C21H20O11	447.0927	447.0939	−2.84	-	-	5.00	-	-	1.50	1.93	2.81
3-Caffeoyl quinic acid	C16H18O9	353.0872	353.0888	−4.80	-	-	4.47	0.96	-	1.34	0.52	0.21
5-p-Coumaroyl quinic acid	C13H16O9	337.0923	337.0940	−5.35	-	-	0.40	7.75	-	0.12	0.12	0.54
Rutin	C27H30O16	609.1455	609.1456	−0.17	0.04	0.05	3.93	11.93	0.39	0.28	0.08	0.45
4-Feruloyl quinic acid	C17H20O9	367.1029	367.1038	−2.60	0.05	0.06	5.97	17.08	0.19	0.67	0.61	0.83
3,4-Dicaffeoyl quinic acid	C25H24O12	515.1189	515.1201	−2.47	-	0.01	4.85	1.69	-	1.46	1.75	0.45
Quercetin-3-O-glucuronide	C21H18O13	477.0669	477.0687	−4.00	-	0.01	0.75	2.36	-	0.22	0.46	0.12
Isorhamnetin 3-O-rhamnoglucoside	C28H32O16	623.1612	623.1614	−0.34	-	0.07	0.74	5.20	0.22	0.22	0.14	0.19
Demethyl hedysarimpterocarpene-A rutinoside	C28H32O15	607.1663	607.1661	0.35	-	0.01	8.25	1.46	0.03	2.48	3.06	0.14
Taxifolin hexoside	C21H22O12	465.1033	465.1044	−2.51	-	0.07	2.18	0.22	0.21	0.65	0.62	0.15
3,5-Dicaffeoyl quinic acid	C25H24O12	515.1189	515.1191	−0.41	-	0.05	0.26	2.54	0.15	0.08	0.02	0.16
4,5-Dicaffeoyl quinic acid	C25H24O12	515.1189	515.1191	−0.41	-	0.08	0.48	4.37	1.74	0.14	0.06	0.16
Taxifolin	C15H12O7	303.0504	303.0521	−5.95	0.23	0.17	0.26	1.40	5.02	0.97	0.03	0.02
3-Caffeoyl-4-feruloyl quinic acid	C26H26O12	529.1346	529.1369	−4.61	0.12	0.20	0.48	0.74	1.58	0.04	0.02	0.04
Isorhamnetin derivative	-	-	777.5536	-	0.14	0.14	0.24	0.56	1.16	0.05	0.05	0.02
p-Hydroxy-benzoic acid	C7H6O3	137.0238	137.0245	−5.42	0.05	0.39	0.12	0.17	1.16	0.04	0.02	0.02
Vanilic acid glucoside	C14H18O9	329.0872	329.0885	−4.20	0.02	0.08	0.10	0.11	1.52	0.03	0.02	0.02
				total	0.65	1.48	41.52	58.82	13.37	10.57	14.82	6.97

* *m*/*z* values refer to [M − H]^−^ ions.

**Table 4:** Content of tannins identified and quantified in the extracts, expressed as mg/g on dry extract. MS data were acquired in negative ion mode (ESI(−)).

					Aerial				Roots			
Tannins	Formula (Neutral Compound)	Theorical *m*/*z* *	Exp *m*/*z* *	ppm	DCM	EA	MeOH	W	DCM	EA	MeOH	W
Ellagic acid hexoside	C20H16O13	465.0669	465.0681	−2.74	-	-	-	-	3.72	3.59	0.01	1.80
Ellagic acid hexoside	C20H16O13	465.0669	465.0683	−3.19	-	-	0.85	-	3.59	2.28	2.56	2.42
Ellagic acid hexoside	C20H16O13	465.0669	465.0681	−2.74	-	-	-	0.35	22.63	0.92	0.01	0.47
Ellagic acid deoxyhexoside	C20H16O12	449.0720	449.0732	−2.83	-	-	0.59	-	2.91	2.84	1.76	2.30
Ellagic acid deoxyhexoside	C20H16O13	449.0721	449.0732	−2.70	-	-	-	-	1.49	1.30	0.02	0.66
Ellagic acid deoxyhexoside	C20H16O14	449.0720	449.0732	−2.80	-	-	-	-	9.46	8.55	0.01	4.28
Ellagic acid derivative	C20H13O14	477.0295	477.0305	−2.22	-	-	0.29	0.64	3.61	3.26	54.86	29.06
Ellagic acid derivative	C20H13O14	477.0295	477.0305	−2.22	-	-	4.27	4.44	1.61	1.48	12.82	7.15
Ellagic acid derivative	C20H13O14	477.0295	477.0305	−2.22	-	-	2.11	1.87	2.31	2.11	6.32	4.21
Ellagic acid	C14H6O8	300.9983	300.9985	−0.56	-	-	5.42	3.74	11.21	10.16	16.25	13.21
				total	-	-	13.53	11.03	62.55	36.49	94.63	65.56

* *m*/*z* values refer to [M + H]^+^ ions.

**Table 5:** Content of coumarins identified and quantified in the extracts, expressed as mg/g on dry extract. MS data were acquired in positive ion mode (ESI(+)).

					Aerial				Roots			
	Formula (Neutral Compound)	Theorical *m*/*z* *	Exp *m*/*z* *	ppm	DCM	EA	MeOH	W	DCM	EA	MeOH	W
**Fraxidin glucuronide ^§^**	C16H16O11	383.0609	383.0614	−1.39	0.004	0.01	1.52	5.66	-	0.46	0.46	0.23
Columbianetin	C14H14O4	247.0970	247.0957	5.62	0.05	0.23	0.28	0.03	-	-	-	-
Columbianidin	C19H20O5	329.1389	329.1407	−5.80	2.46	0.31	1.05	0.39	0.02	0.01	0.09	0.03
Dalbergin isomer	C16H12O4	269.0814	269.0830	−6.30	1.49	0.18	0.66	0.23	-	-	-	-
Marmesin	C14H14O4	247.0970	247.0957	5.62	2.11	0.26	0.98	0.33	-	-	-	-
Oxypeucedanin hydrate	C16H16O6	305.1025	305.1039	−4.86	0.54	0.06	0.27	0.08	-	-	-	-
Isoimperatorin	C16H14O4	271.0970	271.0974	−1.56	1.23	0.15	0.59	0.18	-	-	-	-
8-Methoxypsoralene	C12H8O4	217.0501	217.0521	−9.77	13.19	1.64	6.32	2.08	-	-	-	-
Bergapten	C12H8O4	217.0501	217.0521	−9.77	4.07	0.46	1.68	0.58	0.07	-	-	-
Isopimpinellin 5,8-dimethoxypsoralene	C13H10O5	247.0610	247.0630	−8.58	3.79	0.45	1.62	0.57	0.04	-	-	-
Dalbergin	C16H12O4	269.0814	269.0830	−6.30	1.31	0.15	0.53	0.19	0.09	-	-	-
Angenomalin/isoangenomalin	C14H12O3	229.0845	229.0855	−4.63	4.12	0.41	1.52	0.52	-	-	-	-
Oxypeucedanin	C16H14O5	287.0920	287.0943	−8.49	2.96	0.28	1.03	0.36	-	-	-	-
Xantotoxol	C11H6O4	203.0344	203.0360	−8.35	59.12	11.45	43.74	14.48	-	-	-	-
Deltoin	C19H20O5	329.1389	329.1389	0	2.02	0.17	0.66	0.22	-	-	-	-
Decursin	C19H20O5	329.1389	329.1404	−4.83	30.53	9.29	41.31	11.75	-	-	-	-
Angelomalin/isoangelomalin	C14H12O3	229.0865	229.0885	−9.25	3.77	0.36	1.30	0.45	-	-	-	-
				Total	132.77	25.86	105.04	38.09	0.22	0.47	0.56	0.26

* *m*/*z* values refer to [M + H]^+^ or [M − H]^−^ ions; ^§^ MS data were acquired in negative ion mode (ESI(−)).

**Table 6:** Antioxidant properties of *Sartoria hedysaroides* extracts *.

	Solvents	DPPH (mg TE/g)	ABTS (mg TE/g)	CUPRAC (mg TE/g)	FRAP (mg TE/g)	MCA (mg EDTAE/g)	PBD (mmol TE/g)
Aerial parts	DCM	4.93 ± 0.28 ^h^	31.75 ± 1.31 ^f^	68.71 ± 1.28 ^e^	23.24 ± 0.42 ^f^	15.77 ± 0.21 ^c,d^	1.82 ± 0.08 ^a,b^
	EA	12.46 ± 0.42 ^g^	47.99 ± 0.47 ^e^	92.47 ± 0.82 ^c^	33.04 ± 0.49 ^e^	17.60 ± 0.52 ^c^	2.08 ± 0.11 ^a^
	MeOH	169.97 ± 1.32 ^a^	285.33 ± 11.94 ^a^	279.23 ± 0.20 ^a^	162.48 ± 2.52 ^a^	11.87 ± 0.36 ^d,e^	1.87 ± 0.13 ^a,b^
	Infusion	74.32 ± 1.30 ^b^	164.37 ± 1.62 ^b^	171.80 ± 1.63 ^b^	121.87 ± 3.70 ^b^	23.44 ± 0.21 ^a,b^	1.08 ± 0.01 ^d^
Roots	DCM	25.50 ± 0.25 ^e^	54.06 ± 1.76 ^e^	67.67 ± 0.73 ^e^	36.11 ± 0.44 ^e^	18.99 ± 4.67 ^b,c^	1.68 ± 0.04 ^b,c^
	EA	30.72 ± 0.20 ^d^	71.03 ± 0.79 ^d^	92.58 ± 1.23 ^c^	44.63 ± 0.15 ^d^	9.76 ± 1.57 ^e^	1.73 ± 0.13 ^b^
	MeOH	37.69 ± 0.30 ^c^	99.65 ± 0.38 ^c^	88.63 ± 0.99 ^d^	54.68 ± 1.55 ^c^	11.76 ± 0.36 ^d,e^	1.40 ± 0.19 ^c^
	Infusion	14.79 ± 0.08 ^f^	80.26 ± 1.06 ^d^	59.09 ± 1.65 ^f^	41.41 ± 0.24 ^d^	26.67 ± 0.21 ^a^	1.07 ± 0.04 ^d^

* Values are reported as means ± SD. DCM: Dicholoromethane; EA: Ethyl acetate; MeOH: Methanol; TE: Trolox equivalent; EDTAE: EDTA equivalent; MCA: Metal chelating ability; PBD: Phosphomolybdenum. Different superscripts letters (a–h) indicate significant differences in the extracts (*p* < 0.05).

**Table 7:** Enzyme inhibition properties of *Sartoria hedysaroides* extracts *.

	Solvents	AchE İnhibition (mg GALAE/g)	BchE İnhibition (mg GALAE/g)	Tyrosinase İnhibition (mg KAE/g)	Amylase İnhibition (mmol ACAE/g)	Glucosidase İnhibition (mmol ACAE/g)
Aerial parts	DCM	0.68 ± 0.08 ^d^	5.96 ± 0.71 ^a^	56.71 ± 0.34 ^d^	0.95 ± 0.01 ^b,c^	0.79 ± 0.03 ^d^
	EA	1.29 ± 0.03 ^c^	5.45 ± 1.07 ^a^	57.85 ± 0.08 ^d^	1.07 ± 0.06 ^a^	0.83 ± 0.02 ^c,d^
	MeOH	2.52 ± 0.02 ^a^	2.75 ± 0.24 ^b^	74.14 ± 0.19 ^a^	1.07 ± 0.04 ^a^	1.03 ± 0.01 ^a^
	Infusion	0.54 ± 0.02 ^e^	na	7.02 ± 0.74 ^e^	0.10 ± 0.01 ^e^	1.02 ± 0.01 ^a^
Roots	DCM	na	6.03 ± 0.51 ^a^	56.74 ± 0.07 ^d^	1.04 ± 0.06 ^a,b^	0.90 ± 0.01 ^b^
	EA	1.79 ± 0.09 ^b^	5.14 ± 0.38 ^a^	59.48 ± 1.27 ^c^	1.08 ± 0.04 ^a^	0.87 ± 0.01 ^b,c^
	MeOH	1.73 ± 0.05 ^b^	3.39 ± 0.21 ^b^	64.99 ± 0.12 ^b^	0.87 ± 0.05 ^c^	0.87 ± 0.04 ^b,c^
	Infusion	na	na	1.65 ± 0.03 ^f^	0.26 ± 0.01 ^d^	0.02 ± 0.01 ^e^

* Values are reported as mean ± SD. DCM: Dicholoromethane; EA: Ethyl acetate; MeOH: Methanol; GALAE: Galatamine equivalent; KAE: Kojic acid equivalent; ACAE: Acarbose equivalent; na: not active. Different superscripts letters (a–f) indicate significant differences in the extracts (*p* < 0.05).

**Table 8:** Relationship between total phenolics and flavonoids contents and biological activities (Pearson correlation (R)).

	DPPH	ABTS	CUPRAC	FRAP	MCA	PPBD	AChE	BChE	Tyrosinase	Amylase	Glucosidase
**TPC**	0.97	0.96	0.99	0.96	−0.21	0.08	0.62	−0.36	0.16	−0.02	0.45
**TFC**	0.97	0.95	0.98	0.98	−0.19	0.01	0.52	−0.36	0.13	−0.06	0.54

## Data Availability

Data is contained within the article.

## Supplementary Materials

The following are available online at https://www.mdpi.com/article/10.3390/antiox11010110/s1, All experimental details of the assays are reported in the supplementary material.
